# PRISM: Past‐Regularized Iterative Self‐Distillation With Momentum for Polyp Segmentation

**DOI:** 10.1049/htl2.70050

**Published:** 2025-12-16

**Authors:** Tugberk Erol, Tuba Caglikantar, Duygu Sarikaya

**Affiliations:** ^1^ Graduate School of Natural and Applied Sciences Gazi University Ankara Turkey; ^2^ Department of Software Engineering Faculty of Engineering and Natural Sciences Ankara Yildirim Beyazit University Ankara Turkey; ^3^ School of Computer Science University of Leeds Leeds UK

**Keywords:** convolutional networks, medical image segmentation, polyp segmentation, regularization, self distillation

## Abstract

Polyps are abnormal tissue growths in the colon that may develop into colorectal cancer if left undetected. Accurate segmentation in medical imaging is essential for early diagnosis and treatment. Although deep learning has greatly improved polyp segmentation, its dependence on large annotated datasets and substantial computational resources hampers generalization across diverse clinical settings. To overcome these challenges, we propose PRISM, a momentum‐based self‐distillation method that improves segmentation performance without introducing additional inference cost. Instead of storing or reusing past predictions, PRISM constructs a temporally smoothed teacher model by applying an exponential moving average (EMA) to the model's weights throughout training. This momentum‐based teacher provides stable and adaptive supervision signals that co‐evolve with the student model. We evaluate PRISM on colonoscopy datasets collected from five distinct medical centres and validate its generalization on an unseen independent dataset. PRISM achieves a Dice score of 0.81 and an IoU of 0.75, outperforming baseline and conventional self‐distillation methods. Ablation studies confirm the effectiveness of the EMA‐based teacher model in improving segmentation accuracy. PRISM offers a computationally efficient and generalizable solution for polyp segmentation tasks. The code is available at: https://github.com/TugberkErol/PRISM.

## Graphical abstract

The proposed PRISM method uses momentum‐based self‐distillation with an exponential moving average teacher for polyp segmentation without added inference cost. Tested across diverse colonoscopy datasets, PRISM outperforms conventional methods, achieving a Dice score of 0.81 and IoU of 0.75, demonstrating strong generalization and efficiency.

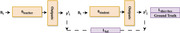

## 1 Introduction

Polyp segmentation in colonoscopy images is a crucial task in early colorectal cancer detection. Polyps, abnormal tissue growths in the colon lining, are potential precursors to colorectal cancer, which makes their accurate detection essential for timely intervention and improved patient outcomes. However, manual identification and segmentation of these polyps can be time‐consuming and are prone to human error, which has driven the need for efficient and reliable automated methods. Recent advances in deep learning have demonstrated great promise in addressing this challenge by enabling precise segmentation of polyps in medical images.

One approach to improving the performance of segmentation models is knowledge distillation (KD), a technique where a smaller, more efficient model (student) learns from a larger, more powerful model (teacher). The teacher model transfers its knowledge, allowing the student to achieve comparable performance with reduced computational cost. This technique is particularly beneficial in medical image analysis, where the deployment of large models for real‐time applications may not always be feasible due to resource constraints.

Self‐distillation (SD), an extension of KD, uses the model itself as both the teacher and the student, where the same model learns from its own predictions [1, 2, 3, 4, 5]. This technique improves the robustness of the model, leading to better generalization and performance, even in the absence of a separate teacher model. Although self‐distillation methods have proven effective in improving segmentation performance, they have notable limitations. One major drawback is their sensitivity to outdated or suboptimal predictions, particularly when these predictions are used as targets for future learning steps. This can negatively influence training, especially when the model is still in early or unstable phases of learning. Additionally, several self‐distillation methods rely on information from the previous mini‐batch or previous epoch, assuming that past outputs provide reliable supervision. However, in medical datasets where data distributions vary significantly across samples or institutions, such assumptions may not hold. These stale or poorly calibrated predictions can lead to inaccurate guidance, which in turn hinders convergence and degrades model performance.

To address these challenges, we propose a robust and adaptive mechanism: PRISM (past‐regularized iterative self‐distillation with momentum). PRISM refines predictions by maintaining an exponential moving average (EMA) of the model weights instead of using only instantaneous or batch‐level predictions. This EMA‐based teacher model captures longer‐term knowledge accumulated across training iterations, providing a more stable and temporally consistent supervision signal. Instead of using only recent predictions, PRISM leverages the predictions of the EMA‐smoothed teacher model, which inherently emphasizes more recent and confident representations while reducing the influence of earlier, potentially noisy states. This temporal smoothing mechanism mitigates the impact of outdated or unstable predictions and ensures that the supervisory signal evolves in alignment with the model learning trajectory.

Furthermore, PRISM integrates a temperature‐scaled sigmoid activation during the distillation process, which softens the output distributions. This not only enhances the model's ability to capture nuanced semantic structures but also produces smoother and more informative targets, facilitating improved representation learning. Through this dual strategy —EMA‐based teacher model construction and temperature‐scaled supervision—PRISM effectively addresses the limitations of conventional self‐distillation. It avoids over‐reliance on noisy or unstable outputs and reduces the variance introduced by single‐step predictions, resulting in more stable and accurate training dynamics.

**FIGURE 1:**
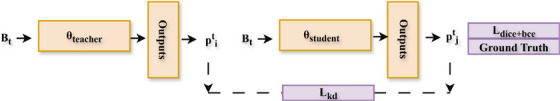
Overview of the polyp segmentation model trained with the PRISM method. The model processes the same input ($B_{t}$) using two parameter sets: the student ($\theta_{\text{student}}$) and the teacher ($\theta_{\text{teacher}}$). Both generate predictions ($p_{j}^{t}$ and $p_{i}^{t}$), and the loss ($L_{kd}$) is computed between them. The student parameters are updated using the computed loss, while the teacher parameters are obtained by a momentum‐weighted combination of the student and teacher parameters.

To evaluate the effectiveness of our proposed technique, we trained the model on the datasets data_c1 to data_c5 and tested it on the independent data_c6 dataset, which features a distinct data distribution. Additionally, we conducted ablation studies to isolate the contributions of each component in the method. The results demonstrate that PRISM not only achieves higher segmentation accuracy but also maintains consistent performance across diverse datasets, showcasing its robustness and generalizability. Through this approach, our goal is to contribute to the advancement of automated polyp detection systems, offering a more reliable and computationally efficient solution for clinical applications, especially in settings where consistent data quality and availability cannot be guaranteed. An overview of the proposed approach is illustrated in Figure 1.

## 2 Related Works

Polyp segmentation has been a crucial task in medical image analysis, with numerous deep learning‐based methods developed over the years. The most widely adopted baseline model, U‐Net [6], introduced an encoder–decoder structure with skip connections to retain both low and high‐level features, making it highly effective for medical image segmentation. Building upon U‐Net, several advanced architectures have been proposed to improve segmentation performance.

HarDNet‐MSEG [7] introduces an efficient encoder–decoder network based on the HarDNet backbone, offering a strong balance between segmentation accuracy and computational speed for polyp detection. PraNet [8] proposed a parallel reverse attention network that leverages a parallel partial decoder to reduce the number of parameters while using reverse attention to capture structural details more effectively in polyp segmentation. More recent approaches, such as ShallowNet [9] and TransNetR [10], have further advanced polyp segmentation by enhancing efficiency and feature representation, with ShallowNet focusing on lightweight architectures and TransNetR incorporating transformer‐based modules.

Several state‐of‐the‐art methods, including HardNet‐MSEG [7], TransNetR [10], ShallowNet [9], ResNetUNet [11], DeepLabV3+ [12], and PraNet [8], have been benchmarked for polyp segmentation. While these models achieve strong results, they still face challenges in generalization, robustness, and handling complex polyp structures.

Knowledge distillation [13] addresses some of these limitations by transferring knowledge from a more powerful teacher model to a student model, improving feature representation and segmentation performance, particularly in challenging cases. For instance, Qin et al. [14] proposed a method for CT liver segmentation where a teacher model distils feature and prediction level knowledge to a student model, improving segmentation performance while reducing computational complexity.

However, recent advancements have shifted focus towards self‐distillation, a variant where a model distils knowledge from its own predictions rather than relying on an external teacher. Ye et al. [15] proposed a deep self‐distillation method that improves segmentation performance by refining feature representations at both shallow and deep layers. This approach leverages self‐generated soft targets, enabling the model to progressively learn more accurate and robust features for 3D medical image segmentation tasks. Similarly, Shen et al. [1] proposed leveraging soft targets from the previous mini‐batch to improve consistency and generalization. These approaches highlight the potential of self‐distillation in refining model representations and improving segmentation performance.

Momentum‐based representation learning and knowledge distillation have been explored in methods like MoCo by He et al. [16], which uses momentum contrastive learning, and graph‐driven momentum distillation (GMoD) by Xiang et al. [17], which applies momentum to graph‐based tasks to improve model stability and performance.

Conventional self‐distillation methods face challenges, including inefficiency and instability when relying solely on the most recent mini‐batch. They may also suffer from outdated data issues, where earlier iterations of the model do not represent the current data distribution, hindering effective knowledge transfer. To address these challenges, we propose a novel momentum‐based self‐distillation approach. Instead of relying solely on the predictions from the most recent mini‐batch, our method employs a momentum‐updated model as a temporal teacher that gradually accumulates knowledge across training iterations. This enables the model to benefit from a richer and more consistent learning signal aggregated over time.

We evaluate the effectiveness of our approach through comparisons with state‐of‐the‐art methods and an ablation study on the data_C6 dataset. The results demonstrate that our method achieves superior performance compared to baseline and conventional self‐distillation methods, leading to improved segmentation accuracy and more coherent learning behaviour.

## 3 Method

Conventional self‐distillation enables a model to learn from its own softened predictions but typically captures only short‐term information from recent outputs. To overcome this limitation, we first introduce the temperature‐scaled sigmoid function and the self‐distillation framework.

The standard sigmoid function maps a real‐valued input x∈R to the interval (0,1) as:

(1) $$\sigma \left(x\right)=\frac{1}{1+e^{-x}}.$$

**FIGURE 2:**
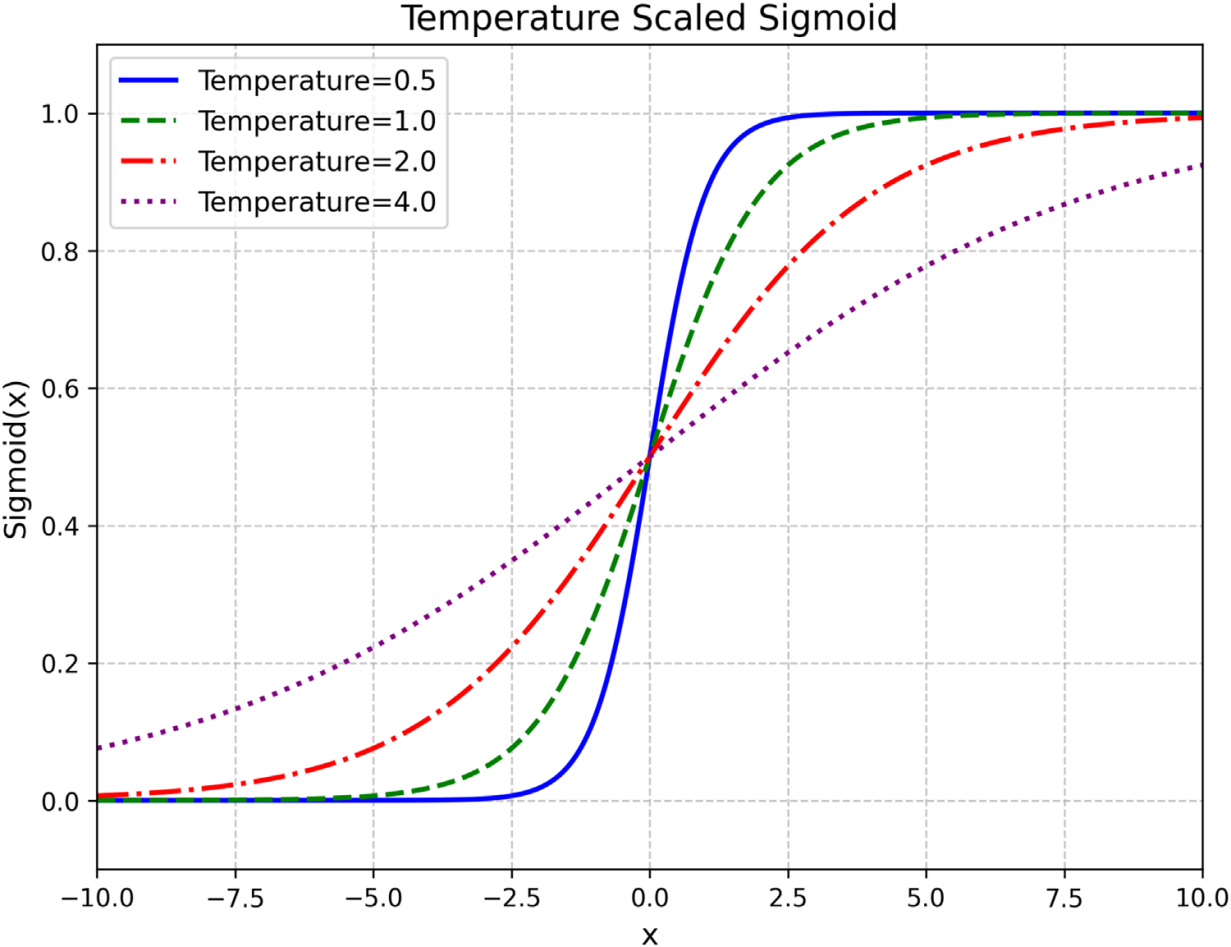
Effect of temperature scaling on the sigmoid function. Lower temperatures (e.g., T=0.5) yield sharper, more binary‐like transitions, while higher temperatures (e.g., T=4.0) produce smoother outputs.

To adjust the output sharpness, we use a temperature‐scaled version:

(2) $$\sigma_{T}\left(x\right)=\frac{1}{1+e^{-x/T}},$$

where T>0 is the temperature parameter. Lower values of T produce sharper, more binary‐like outputs, while higher values result in softer predictions (see Figure 2).

This temperature scaling offers two main advantages: (i) it acts as a regularizer that mitigates early overconfidence in predictions, and (ii) it provides a tuneable balance between hard and soft targets, which is particularly useful in self‐distillation.

Self‐distillation is a training technique where a model refines its own predictions by learning from softened versions of its previous outputs, without requiring an external teacher model. Given an input X, the soft target at iteration t is computed as:

(3) $$P_{s}^{\left(t\right)}=\sigma_{T}\left(f_{\theta }\left(X\right)\right)=\sigma \left(\frac{f_{\theta }\left(X\right)}{T}\right),$$

where $f_{\theta }\left(X\right)$ denotes the model's logits and T is the temperature controlling output smoothness.

The self‐distillation loss encourages consistency between successive soft targets and is defined as the mean squared error (MSE) between predictions at consecutive iterations:

(4) $$L_{\text{sd}}=\text{MSE}\left(P_{s}^{\left(t\right)},P_{s}^{\left(t-1\right)}\right).$$

The overall loss combines supervised learning with self‐distillation:

(5) $$L=L_{\text{sup}}+\lambda L_{\text{sd}},$$

where $L_{\text{sup}}$ is the standard supervised loss and λ balances the distillation contribution. This formulation helps stabilize training and improve model generalization by leveraging its own temporally smoothed predictions as guidance.

### 3.1 PRISM: Past‐Regularized Iterative Self‐Distillation With Momentum

To address the limitations of conventional self‐distillation methods, which often rely solely on immediate predictions from individual mini‐batches, we propose PRISM. This method introduces a momentum‐based strategy that accumulates knowledge across multiple training iterations, enabling the model to progressively refine its internal representations.

Inspired by momentum‐based optimization techniques, PRISM leverages an EMA of model weights to construct a temporally smoothed teacher model, which provides stable and informative supervision for distillation. Specifically, at each iteration t, the teacher model's parameters are updated as:

(6) $$\theta_{\text{teacher}}^{\left(t\right)}=\gamma \theta_{\text{teacher}}^{\left(t-1\right)}+\left(1-\gamma \right)\theta_{t},$$

where γ is the momentum coefficient and $\theta_{t}$ represents the student model's parameters.

The total loss function in PRISM combines Dice loss, binary cross‐entropy loss and knowledge distillation loss from the momentum‐smoothed teacher outputs:

(7) $$L_{t}=L_{\text{Dice}}\left(\hat{y},y\right)+L_{\text{BCE}}\left(\hat{y},y\right)+\lambda L_{\text{KD}}\left({\hat{y}}_{\text{soft}},{\tilde{y}}_{\text{soft}}\right),$$

where $\hat{y}$ and $\tilde{y}$ denote the student and teacher outputs, respectively.

To allow the student model to first learn stable feature representations, PRISM delays momentum‐based distillation until after the sixth training epoch. This ensures that the student begins to incorporate past knowledge when it has already achieved a reasonable feature representation.

Algorithm 1 summarizes the PRISM training procedure, and an architectural overview is shown in Figure 1.

ALGORITHM 1 **PRISM.**

**Table:** 

**Require**: Model parameters θ, learning rate η, momentum factor γ, distillation temperature T, dataset D	
**Ensure**: Optimized model parameters $\theta^{*}$	
1:	Initialize model parameters $\theta_{0}$
2:	Initialize teacher model parameters $\theta_{\text{teacher}}=\theta_{0}$
3:	**for** each training iteration t=1,⋯,T **do**
4:	Sample mini‐batch (x,y)∼D
5:	Compute student model prediction: $\hat{y}\leftarrow f_{\theta_{t}}\left(x\right)$
6:	Compute teacher soft target: $\tilde{y}\leftarrow \sigma \left(f_{\theta_{\text{teacher}}}\left(x\right)/T\right)$
7:	Compute total loss: $L_{t}=L_{\text{Dice}}\left(\hat{y},y\right)+L_{\text{BCE}}\left(\hat{y},y\right)+\lambda L_{\text{KD}}\left({\hat{y}}_{soft},{\tilde{y}}_{soft}\right)$
8:	Compute gradient: $g_{t}\leftarrow \nabla_{\theta }L_{t}$
9:	Update student model parameters: $\theta_{t}\leftarrow \theta_{t-1}-\eta g_{t}$
10:	Update teacher model parameters with momentum: $\theta_{\text{teacher}}\leftarrow \gamma \theta_{\text{teacher}}+\left(1-\gamma \right)\theta_{t}$
11:	**end for**
12:	**Return** optimized parameters $\theta_{T}$

## 4 Results

To comprehensively evaluate the performance and generalizability of the proposed PRISM model, we conducted experiments using six publicly available colonoscopy image datasets. Among these, five datasets: data_c1, data_c2, data_c3, data_c4 and data_c5 [18] were employed for training purposes, while the remaining dataset, data_c6 [18], was held out exclusively for evaluation to simulate a real‐world test scenario. These datasets were collected from different clinical centres, providing a diverse and realistic benchmark for assessing the model's robustness. The characteristics of each dataset, such as the number of images and labelling details, are summarized in Table 1.

**TABLE 1:** Datasets used to evaluate our model on as well as the sites where they were collected.

Dataset	#images	Type	Centre
data_c1	256	Training	Ambroise Paré Hospital (Paris)
data_c2	301	Training	Istituto Oncologico Veneto (Padova)
data_c3	457	Training	Centro Riferimento Oncologico (IRCCS)
data_c4	227	Training	Oslo University Hospital (Oslo)
data_c5	208	Training	John Radcliffe Hospitals (Oxford)
data_c6	88	Testing	University of Alexandria (Alexandria, Egypt)

### 4.1 Experimental Setup

For training, we adopted an end‐to‐end optimization strategy using the AdamW optimizer [19], with a fixed learning rate of $1\times 10^{-4}$, a batch size of 4, and a momentum parameter set to 0.90. All input images across datasets were resized to 256×256 pixels to maintain consistency in spatial dimensions and to ensure compatibility with the model architecture. The dataset was split into 90% for training and 10% for validation. During training, model checkpoints were saved based on the lowest validation loss to ensure optimal generalization performance. The training process spanned 30 epochs, which was empirically found to be sufficient for convergence without overfitting.

### 4.2 Comparison With State‐of‐the‐Art Methods

To benchmark the effectiveness of our approach, we compared PRISM against several state‐of‐the‐art (SOTA) segmentation methods that had demonstrated strong performance in medical image analysis. These included FCN [20], U‐Net [6], PSPNet [21], ResNetUNet (ResNet34 backbone) [11], DeepLabV3+ (with ResNet50) [12, 22], HarDNet‐MSEG [7], ShallowNet [9], TransNetR [10], and PraNet [8]. These models served as competitive baselines for evaluating the effectiveness of the proposed momentum‐based distillation strategy employed in PRISM.

Quantitative results of all models are reported in Table 2, where PRISM consistently outperformed the other approaches across multiple evaluation metrics. Specifically, our model achieved a Dice score of 0.81, Intersection over Union (IoU) of 0.75, Precision of 0.93, and Recall of 0.80, highlighting both its segmentation accuracy and reliability. The superior performance of PRISM underscored the benefit of integrating momentum‐based self‐distillation for enhancing feature representation and guiding more accurate predictions.

**TABLE 2:** Performance comparison of different segmentation models. The models are evaluated using Dice coefficient, IoU, Precision and Recall metrics. The proposed PRISM method, when integrated with the PraNet model, improves overall performance, achieving the highest Dice, IoU, Precision and Recall scores.

Method	Dice	IoU	Precision	Recall
FCN [20]	0.76	0.68	0.90	0.74
U‐Net [6]	0.63	0.55	0.76	0.66
TransNetR [10]	0.72	0.66	**0.93**	0.70
ShallowNet [9]	0.76	0.70	**0.93**	0.77
HarDNet‐MSEG [7]	0.77	0.70	0.88	0.78
PSPNet [21]	0.80	0.72	0.88	0.79
ResNetUNet(ResNet34) [11]	0.79	0.73	0.92	0.78
DeepLabV3+(ResNet50) [12]	**0.81**	**0.75**	0.92	0.79
Pranet [8]	0.78	0.72	0.92	0.79
Pranet + PRISM (Ours)	**0.81**	**0.75**	**0.93**	**0.80**

### 4.3 Ablation Study

We conducted an ablation study to assess the impact of different training strategies for the performance of the segmentation model. Specifically, we compared three configurations: the base model (Base) trained without any distillation, the conventional self‐distillation (SD) method where the model learned from its own softened predictions, and the proposed PRISM method, which incorporated momentum‐based updates during the distillation process.

**FIGURE 3:**
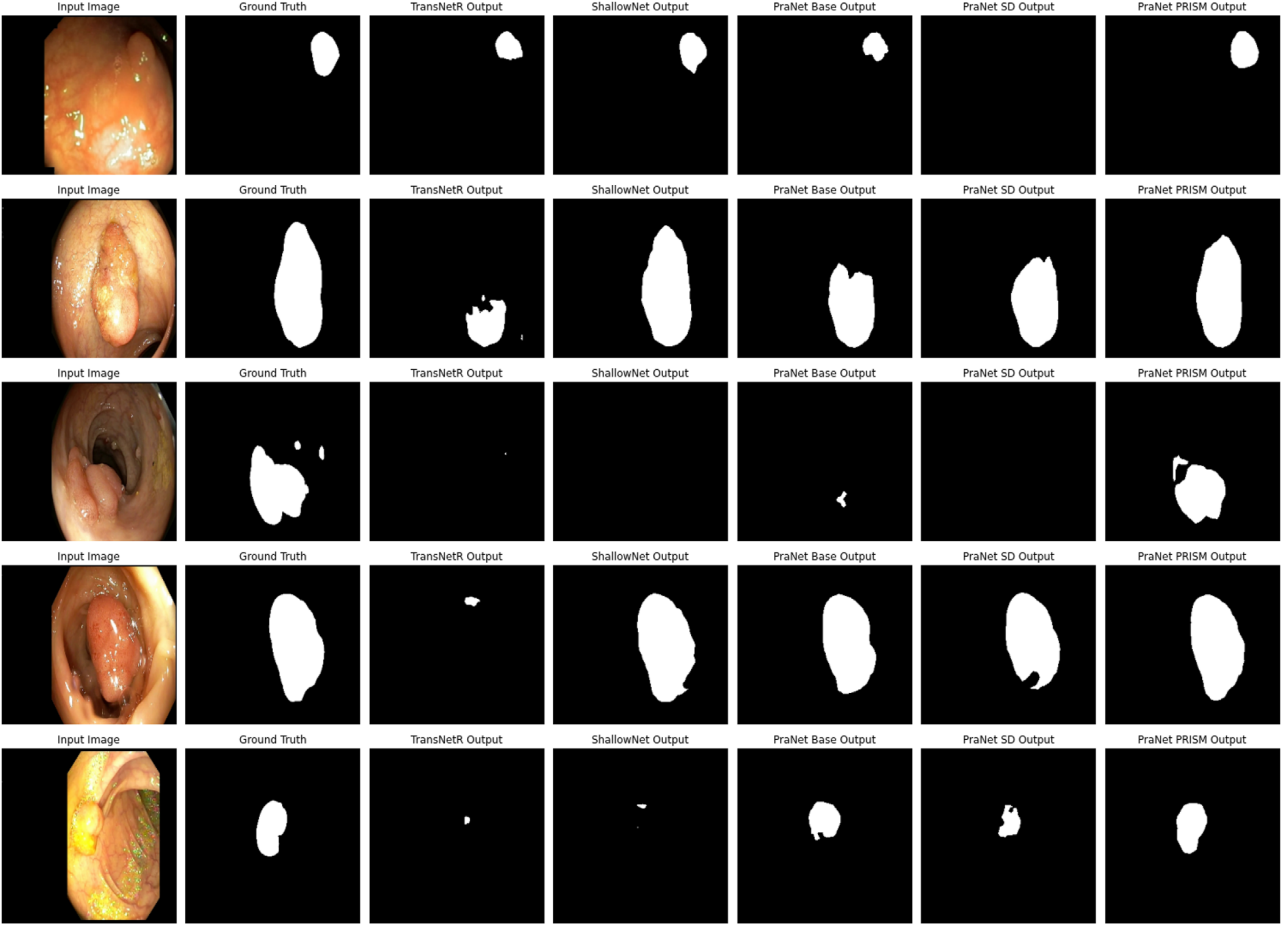
Comparison of model outputs from TransNetR, ShallowNet, PraNet, PraNet with SD, and PraNet with PRISM, highlighting their differences in segmentation performance on the data_c6 dataset.

Experimental results demonstrated that the proposed PRISM method achieved the highest performance among the three approaches. In particular, PRISM obtained a Dice score of 0.809 on the data_c6 dataset, outperforming both the base model and the conventional self‐distillation approach. These results suggested that the momentum‐based strategy used in PRISM contributed positively to the learning process by providing more stable and informative supervisory signals during training. A detailed summary of the results is presented in Table 3, where all key metrics, including Dice, IoU, Precision and Recall, are reported for each method. Additionally, visual comparisons of segmentation outputs are illustrated in Figure 3, showcasing the qualitative differences between the three approaches. The table and figure together highlighted the consistent advantage of PRISM over its counterparts.

**TABLE 3:** Quantitative comparison of the proposed PRISM method against the baseline (Base) and conventional self‐distillation (SD) methods, evaluated using Dice, IoU, Precision, and Recall metrics. The results demonstrate that our method, PRISM, consistently improves segmentation performance across all state‐of‐the‐art models for Dice and IoU metrics.

Method	Dice	IoU	Precision	Recall
*TransNetR*				
Base	0.7189 ± 0.3672	0.6622 ± 0.3555	**0.9348** ± 0.1577	0.6952 ± 0.3708
Self‐distillation (SD)	0.7413 ± 0.3322	0.6745 ± 0.3287	0.9129 ± 0.1689	0.7310 ± 0.3400
PRISM	**0.7615** ± 0.3133	**0.6934** ± 0.3147	0.8951 ± 0.1959	**0.7663** ± 0.3169
*ShallowNet*				
Base	0.7637 ± 0.3285	0.7030 ± 0.3248	**0.9253** ± 0.1258	0.7684 ± 0.3375
Self‐distillation (SD)	0.7696 ± 0.3146	0.7036 ± 0.3096	0.9048 ± 0.1649	0.7891 ± 0.3127
PRISM	**0.7874** ± 0.3030	**0.7229** ± 0.2986	0.9025 ± 0.1789	**0.7979** ± 0.2987
*PraNet*				
Base	0.7824 ± 0.3047	0.7181 ± 0.3060	0.9151 ± 0.1698	0.7896 ± 0.2977
Self‐distillation (SD)	0.8036 ± 0.2864	0.7396 ± 0.2877	**0.9312** ± 0.1125	**0.7985** ± 0.2966
PRISM	**0.8093** ± 0.2806	**0.7451** ± 0.2817	0.9263 ± 0.1433	0.7973 ± 0.2899

Through this comprehensive evaluation pipeline, we demonstrated that PRISM delivered state‐of‐the‐art performance and maintained robustness across different colonoscopy datasets.

### 4.4 Statistical Significance and Variance Analysis

The Wilcoxon signed‐rank test results presented in Table 4 compare the baseline, self‐distillation, and PRISM variants across three architectures: TransNetR, ShallowNet, and PraNet. The results indicate that PRISM achieves statistically significant improvements over both the baseline and self‐distillation models in the TransNetR architecture (p<0.05 for Dice, IoU and Recall). This suggests that the observed gains are unlikely due to random variation and instead reflect a consistent and meaningful enhancement in segmentation performance.

**TABLE 4:** Statistical significance analysis (Wilcoxon signed‐rank test) for Dice, IoU, Precision, and Recall across Base, self‐distillation (SD), and PRISM.

Architecture	Metric	Base vs SD (p)	SD vs PRISM (p)	Base vs PRISM (p)
TransNetR	Dice	0.95	0.02	0.03
	IoU	0.91	0.03	0.04
	Precision	0.04	0.29	0.02
	Recall	0.36	0.00	0.00
ShallowNet	Dice	0.38	0.21	0.33
	IoU	0.36	0.19	0.38
	Precision	0.50	0.91	0.81
	Recall	0.58	0.10	0.46
PraNet	Dice	0.30	0.59	0.39
	IoU	0.34	0.54	0.38
	Precision	0.33	0.06	0.10
	Recall	0.21	0.09	0.48

For ShallowNet and PraNet, the p‐values for Dice and IoU generally fall in the range of approximately 0.2–0.4, indicating that the performance improvements do not reach the conventional significance threshold of 0.05. However, as shown in Table 3, PRISM consistently demonstrates lower standard deviations in Dice scores across all three architectures compared to both baseline and self‐distillation methods.

Considering the test set differs substantially from the training set in terms of image distribution and visual characteristics, thereby introducing a domain shift scenario known to challenge model generalization, high performance variance typically indicates reduced robustness. In contrast, PRISM's lower variance highlights its ability to produce more stable and reliable predictions, even when statistical significance is not always achieved. This stability is particularly critical in medical image segmentation, where both peak accuracy and prediction consistency directly impact clinical reliability and decision‐making.

## 5 Conclusion

Polyps are abnormal tissue growths that can develop in the colon and are considered potential precursors to colorectal cancer. Early detection and accurate segmentation of polyps play a critical role in preventing cancer progression and improving patient outcomes. In this study, we introduce PRISM, a momentum‐based distillation method designed to improve knowledge transfer by maintaining an EMA of the model weights. This strategy enables the construction of a temporally smoothed teacher model whose predictions guide the student with improved stability. By incorporating temporal consistency into the distillation process, PRISM allows the model to more effectively learn from both hard and soft targets, thereby improving overall segmentation performance. We integrate PRISM into three state‐of‐the‐art polyp segmentation architectures PraNet, TransNetR and ShallowNet, and evaluate its effectiveness on the data_c6 [18] colonoscopy dataset. Experimental results show that PraNet augmented with PRISM achieves superior segmentation accuracy and outperforms a range of existing methods, including FCN, U‐Net, PSPNet, ResNetUNet, DeepLabV3+, ShallowNet, TransNetR and HarDNet‐MSEG, across standard metrics such as Dice score, IoU, Precision and Recall. Additionally, ablation studies confirm that PRISM consistently outperforms both the baseline model (without distillation) and the conventional self‐distillation approach, underscoring the benefit of momentum‐guided knowledge accumulation through weight‐based EMA. One practical consideration observed during experimentation is the sensitivity of PRISM to the momentum coefficient, which plays a key role in determining the quality and stability of the distilled knowledge throughout training. Furthermore, a failure case analysis suggests that the effectiveness of PRISM also depends on the design of the distillation loss function. Improper weighting or suboptimal formulation of this loss may lead to unstable training behaviour or diminish the distillation signal's impact on the student. These insights indicate promising directions for future work, such as adaptive momentum scheduling strategies or the development of hybrid distillation objectives tailored to the challenges of medical image segmentation.

## Author Contributions

**Tugberk Erol**: methodology, visualization, software, writing. **Tuba Caglikantar**: methodology, supervision, writing, review and editing. **Duygu Sarikaya**: methodology, visualization, supervision, writing, review and editing.

## Funding

The authors have nothing to report.

## Conflicts of Interest

The authors declare no conflicts of interest.

## Data Availability

All data used in this work is publicly available. The dataset can be accessed at the following link: https://www.synapse.org/Synapse:syn45200214; see also: https://www.nature.com/articles/s41597‐023‐01981‐y.
